# Supplementary material for: Retinal Disease Variability in Female Carriers of RPGR Variants Associated with Retinitis Pigmentosa: Clinical and Genetic Parameters
Source: Genes (Basel). 2025 Feb 13;16(2):221. doi: 10.3390/genes16020221 (PMC11855607; doi:10.3390/genes16020221)
Supplement: Supplementary file 1 [file genes-16-00221-s001.zip › Supplementary Table S1_Summary of clinical tests Tracked.pdf]

Table S1. Summary of the clinical tests performed. Full protocol has been published previously[18]

Clinical Tests	Testing conditions	Outcomes
Participant clinical history	• Interview	○ Demographics<br>○ Any known systemic and/or ocular conditions<br>○ Family ocular conditions, particularly IRDs
Subjective refraction	• ETDRS letter chart<br>• Room lights off<br>• 2.0 log unit neutral density filter for LLVA	○ Spherical equivalent prescription<br>○ BCVA<br>○ LLVA<br>○ LLD
Anterior eye examination	• Slit lamp microscope	○ Prescence of any corneal and/or lenticular opacities that may have prevented good-quality retinal imaging
Fundus-tracked microperimetry	• MAIA microperimetry<br>• Prior to pupil dilation<br>• Mesopic test conditions<br>• Macular centred 68-points	○ Average retinal sensitivity threshold<br>○ HoV volume
OCT	• Following pupil dilation<br>• Spectralis, Heidelberg Engineering, Heidelberg, Germany<br>• Melbourne: 30° x 20°, 49 B-scans, High resolution, ART ≥ 9<br>• Perth: 30° x 25°, 61 B-scans, High resolution, ART ≥ 9	○ Inner-retinal thickness<br>○ Photoreceptor complex thickness
Ultrawide retinal fundus photography	• Optos (Optos plc, Dunfermline, Scotland, UK)<br>• Red-green and FAF imaging (centred)	○ Any retinal abnormalities<br>○ Confirmation of retinal phenotype (using FAF)
FAF	• Following pupil dilation<br>• Spectralis, Heidelberg Engineering, Heidelberg, Germany<br>• 30° at both sites, plus 55° in Perth	○ Retinal classification as defined by Nanda et al.[4]

Abbreviations: BCVA, best-corrected visual acuity; ETDRS, Early Treatment Diabetic Retinopathy Study; FAF, fundus autofluorescence; HoV, hill of vision; IRDs, inherited retinal diseases; LLD, low luminance deficit; LLVA, low-luminance visual acuity; MAIA, Macular Integrity Assessment; OCT, optical coherence tomography; UK, United Kingdom
