# Supplementary material for: Retinal Disease Variability in Female Carriers of RPGR Variants Associated with Retinitis Pigmentosa: Clinical and Genetic Parameters
Source: Genes (Basel). 2025 Feb 13;16(2):221. doi: 10.3390/genes16020221 (PMC11855607; doi:10.3390/genes16020221)
Supplement: Supplementary file 1 [file genes-16-00221-s001.zip › Supplementary Table S2_detailed demographics_Clean Tracked.pdf]

Table S2. *RPGR* carriers' demographics in order of variant location

ID	Age	Identified *RPGR* variant	NCBI Reference Sequence	Type of Variant	GnomAD Frequency	Retinal Phenotype (OD)	Retinal Phenotype (OS)	logMAR BCVA (OD)	logMAR BCVA (OS)
M34	26	c.(?_-1)_(28+1_29-1)del	NM_001034853.2 and NM_000328.2	Complete deletion	Not reported	Radial	Radial	-0.02	0.14
M25	44	c.619+5G>A	NM_001034853.2	Splice region	Not reported	Male Pattern	Male Pattern	0.6	0.5
M24	50	c.1218_1219del	NM_001034853.2	Frameshift (Exon 10)	Not reported	Radial	Radial	0.08	0.12
P17[‡]	7	c.1399C>T	NM_000328.2	Frameshift (Exon 11)	Not reported	Focal	Focal	†	†
P21[1]	36	c.2045_2046dupGT	NM_001034853.2	Frameshift (ORF15)	Not reported	Normal	Normal	†	†
P26[1]	55	c.2045_2046dupGT	NM_001034853.2	Frameshift (ORF15)	Not reported	Radial	Radial	0.4	0.1
P33[1]	34	c.2045_2046dupGT	NM_001034853.2	Frameshift (ORF15)	Not reported	Radial	Radial	†	†
P29[‡]	27	c.2236_2237del	NM_001034853.2	Frameshift (ORF15)	0.0004%	Focal	Radial	0	0
M11[2]	58	c.2252_2255del	NM_001034853.2	Frameshift (ORF15)	Not reported	Focal	Focal	0.16	0.1
M32[2]	54	c.2252_2255del	NM_001034853.2	Frameshift (ORF15)	Not reported	Radial	Focal	-0.08	1.02
P35[‡]	17	c.2257_2260delGGAG	NM_001034853.2	Frameshift (ORF15)	Not reported	Radial	Radial	0.06	0.2
P28[‡]	54	c.2280del	NM_001034853.2	Frameshift (ORF15)	Not reported	Focal	Focal	0.16	1.18
M09[3]	51	c.2362_2366del	NM_001034853.2	Frameshift (ORF15)	Not reported	Male Pattern	Male Pattern	3.8	0.5
M35[3]	79	c.2362_2366del	NM_001034853.2	Frameshift (ORF15)	Not reported	Male Pattern	Male Pattern	0.4	0.24
P32[‡]	72	c.2405_2406delAG	NM_001034853.2	Frameshift (ORF15)	0.0004%	Male Pattern	Male Pattern	2.6	0.42
P18[‡]	44	c.2426_2427delAG	NM_001034853.2	Frameshift (ORF15)	Not reported	Radial	Radial	†	†
P34	58	c.2426_2427AG	NM_001034853.2	Frameshift (ORF15)	Not reported	Normal	Radial	0.1	0
M29	47	c.2442_2445del	NM_001034853.2	Frameshift (ORF15)	Not reported	Focal	Focal	0.12	0.04
P23[‡4]	54	c.2442_2445delAGAG	NM_001034853.2	Frameshift (ORF15)	Not reported	Normal	Normal	-0.04	0
P27[4]	34	c.2442_2445del	NM_001034853.2	Frameshift (ORF15)	Not reported	Normal	Normal	-0.08	-0.1
M20[5]	72	c.2620G>T	NM_001034853.2	Nonsense (ORF15)	Not reported	Normal	Normal	0.1	0.34

M21[5]	26	c.2620G>T	NM_001034853.2	Nonsense (ORF15)	Not reported	Radial	Radial	0.44	0.34
P24[6]	26	c.2625dupA	NM_001034853.2	Duplication (ORF15)	Not reported	Focal	Focal	0.4	0.2
P25[6]	21	c.2625dupA	NM_001034853.2	Duplication (ORF15)	Not reported	Radial	Radial	0.12	0.1
P30*	32	c.2625dupA	NM_001034853.2	Duplication (ORF15)	Not reported	Focal	Focal	†	†
M10[7]	31	c.2635delG	NM_001034853.2	Frameshift (ORF15)	Not reported	Radial	Radial	-0.1	-0.02
P19[7]	30	c.2635delG	NM_001034853.2	Frameshift (ORF15)	Not reported	Focal	Focal	0	0
P20[7]	25	c.2635delG	NM_001034853.2	Frameshift (ORF15)	Not reported	Radial	Radial	-0.18	-0.1
P39‡	36	c.2993_2997delAAGGG	NM_001034853.2	Frameshift (ORF15)	0.0001%	Radial	Focal	0.06	1.36
M17	48	c.3302_3306del	NM_001034853.2	Frameshift (ORF15)	Not reported	Normal	Normal	0.1	-0.08
M36[8]	56	Rearrangement: Exons 13-15§	NM_001034853.1 NM_000328 NM_001034853.1	Complex rearrangement and deletion (Exons 13-15)	Not reported	Focal	Focal	0.26	0
M37[8]	80	Rearrangement: Exons 13-15§	NM_001034853.1 NM_000328 NM_001034853.1	Complex rearrangement and deletion (Exons 13-15)	Not reported	Normal	Normal	-0.08	0.02
P22‡	20	?				Focal	Male Pattern	0.24	0.66
P37‡	15	?				Normal	Normal	0.12	0.22
P38‡	21	?				Radial	Radial	0.4	0.2

† No vision documented; ‡ microperimetry not performed; * only ultrawide retinal images were captured; § complex structural rearrangement of exons 13-15 reported in genetic report. [1]P26 is the aunt of both P21 and P33, who are first cousins; [2]M11 and M32 are sisters; [3]M09 is the daughter of M35; [4]P23 is the mother of P27; [5]M20 is the grandmother of M21; [6]P24 and P25 are sisters; [7]M10, P19 and P20 are sisters; [8]M36 is the daughter of M37
