# Supplementary material for: Retinal Disease Variability in Female Carriers of RPGR Variants Associated with Retinitis Pigmentosa: Clinical and Genetic Parameters
Source: Genes (Basel). 2025 Feb 13;16(2):221. doi: 10.3390/genes16020221 (PMC11855607; doi:10.3390/genes16020221)
Supplement: Supplementary file 1 [file genes-16-00221-s001.zip › Supplementary Table S3_clinical tests.pdf]

Table S3. Clinical tests for controls and *RPGR* carriers

P-values represent comparison of *RPGR* carriers with healthy controls.

Abbreviations: BCVA, best-corrected visual acuity; dB, decibels; ETDRS, early treatment diabetic retinopathy study; HoV, hill of vision; IRT, inner retinal thickness; PRC, photoreceptor complex; SD, standard deviation. † Age is a factor affecting the classification-outcome relationship (p<0.05). ‡ Median (IQR) reported

	Mean ± SD Age-adjusted p-value comparing to controls				
	**Normal (n=15 eyes)**	**Radial (n=26 eyes)**	**Focal Pigmentary (n=18 eyes)**	**Male pattern (n=9 eyes)**	**Control (n=60 eyes)**
Refractive error (dioptre)†	-0.63 ± 3.82 *p*=0.881	-1.80 ± 3.51 *p*=0.386	**-4.83 ± 5.30 p=0.011**	-2.66 ± 2.86 *p*=0.076	-0.90 ± 2.25 -
BCVA (logMAR)‡	0.02 (-0.08,0.11) *p*=0.266	0.06 (-0.08, 0.14) *p*=0.122	**0.16 (0.02, 0.33) *p*<0.001**	**0.42 (0.35, 1.63) *p*<0.001**	-0.1 (-0.16, -0.06) -
LLVA (logMAR)‡	0.3 (0.26, 0.34) *p*=0.184	0.18 (0.16, 0.29) *p*=0.312	**0.38 (0.28, 0.54) *p*<0.001**	**0.6 (0.53, 1.06) *p*<0.001**	0.16 (0.1, 0.24) -
LLD (ETDRS letters)‡	11 (10, 19) *p*=0.443	14 (9, 15.5) *p*=0.913	14 (8, 14) *p*=0.268	18 (9, 32) ***p*<0.01**	13 (10, 16) -
Average threshold (dB)†	27.4 ± 2.8 *p*=0.568	27.0 ± 1.2 *p*=0.395	**22.7 ± 3.1 *p*<0.001**	**12.1 ± 9.8 *p*<0.001**	27.1 ± 1.7 -
HoV volume (dB-degrees^2)†	6448.8±637.2 *p*=0.602	6377.9±277.1 *p*=0.375	**5458.2±657.2 *p*<0.01**	**2945.7±2298.4 *p*<0.001**	6401.1±387.6 -
IRT 7° (µm)†	173.4 ± 12.0 *p*=0.727	**170.5 ± 13.7 *p*=0.028**	**174.0 ± 17.8 *p*=0.034**	**148.2 ± 19.3 *p*=0.027**	175.7 ± 11.9 -
IRT 5° (µm)	195.3 ± 11.3 *p*=0.715	194.2 ± 14.9 *p*=0.199	194.1 ± 19.1 *p*=0.112	**155.0 ± 27.0 *p*<0.001**	197.6 ± 11.2 -
IRT 3° (µm)	172.8 ± 12.6 *p*=0.796	178.8 ± 17.2 *p*=0.660	182.8 ± 20.4 *p*=0.246	**145.4 ± 41.0 *p*=0.001**	174.8 ± 12.4 -
IRT 1° (µm)	69.9 ± 13.1 *p*=0.507	79.2 ± 26.1 *p*=0.085	**103.6 ± 33.5 *p*<0.001**	**93.9 ± 45.4 *p*=0.002**	65.7 ± 13.7 -
PRC 7° (µm)	**103.3 ± 7.2 *p*=0.004**	**99.1 ± 9.8 *p*<0.001**	**80.7 ± 17.7 *p*<0.001**	**69.0 ± 18.9 *p*<0.001**	115.3 ± 7.3 -
PRC 5° (µm)	**108.2 ± 8.7 *p*=0.001**	**104.5 ± 10.6 *p*<0.001**	**94.0 ± 19.1 *p*<0.001**	**76.9 ± 22.9 *p*<0.001**	122.6 ± 9.1 -
PRC 3° (µm)	**118.0 ± 12.0 *p*=0.004**	**115.3 ± 12.3 *p*<0.001**	**106.9 ± 20.2 *p*<0.001**	**77.7 ± 30.6 *p*<0.001**	133.9 ± 11.5 -
PRC 1° (µm)	**150.1 ± 18.1 *p*=0.033**	**147.9 ± 18.1 *p*<0.001**	**133.4 ± 28.0 *p*<0.001**	**90.2 ± 31.7 *p*<0.001**	165.6 ± 15.1 -
