# Supplementary material for: Retinal Disease Variability in Female Carriers of RPGR Variants Associated with Retinitis Pigmentosa: Clinical and Genetic Parameters
Source: Genes (Basel). 2025 Feb 13;16(2):221. doi: 10.3390/genes16020221 (PMC11855607; doi:10.3390/genes16020221)
Supplement: Supplementary file 1 [file genes-16-00221-s001.zip › Supplementary Table S4_genetic variant.pdf]

Table S4. Outcome measures compared based on loca6on of gene6c muta6on and healthy controls

Mixed effects models were used to assess the rela3onship between clinical outcomes and gene3c variant loca3on. Abbrevia3ons: BCVA, best-corrected visual acuity; dB, decibels; ETDRS, early treatment diabe3c re3nopathy study; HoV, hill of vision; IRT, inner re3nal thickness; PRC, photoreceptor complex; SD, standard devia3on. † Age is a factor affec3ng the classifica3on-outcome rela3onship (p<0.05). ‡ Median (IQR) reported. ¥ One carrier was not included in the analysis, as no clinical tests were performed, only ultrawide re3nal imaging and genotyping.

	Mean ± SD<br>*p*-value age-adjusted comparing to controls<br>*p*-value age-adjusted comparing to ORF15 carriers		
	ORF15<br>(n=50 eyes)¥	Exons 1-14<br>(n=12 eyes)	Healthy controls<br>(n=60 eyes)
Refractive error (dioptre)†	-2.50 ± 4.75	-2.00 ± 2.75	-0.90 ± 2.25
p-value (controls)	***p*=0.044**	*p*=0.464	-
p-value (ORF15)	-	*p*=0.537	*p*=0.044
BCVA (logMAR)‡	0.1 (0, 0.22)	0.01 (-0.08, 0.26)	-0.1 (-0.16, -0.06)
p-value (controls)	***p*<0.001**	*p*=0.335	-
p-value (ORF15)	-	*p*=0.133	***p*<0.001**
LLVA (logMAR)‡	0.34 (0.26, 0.59)	0.27 (0.16, 0.52)	0.16 (0.1, 0.24)
p-value (controls)	***p*<0.001**	*p*=0.058	-
p-value (ORF15)	-	***p*=0.018**	***p*<0.001**
LLD (ETDRS letters)‡	14 (9, 18.5)	11.5 (9, 14)	13 (10, 16)
p-value (controls)	*p*=0.272	*p*=0.283	-
p-value (ORF15)	-	*p*=0.091	*p*=0.272
Average threshold (dB)†	23.9 ± 7.1	25.1 ± 2.9	27.1 ± 1.7
p-value (controls)	***p*=0.006**	*p*=0.420	-
p-value (ORF15)	-	*p*=0.397	***p*=0.006**
HoV volume (dB-degrees^2)†	5669.1 ± 1623.3	5936.6 ± 656.0	6401.1±387.6
p-value (controls)	***p*=0.006**	*p*=0.428	-
p-value (ORF15)	-	*p*=0.394	***p*=0.006**
IRT 7° (µm)†	169.3 ± 17.5	168.0 ± 17.0	175.7 ± 11.9
p-value (controls)	*p*=0.050	*p*=0.178	-
p-value (ORF15)	-	*p*=0.879	*p*=0.050
IRT 5° (µm)†	187.9 ± 23.0	190.9 ± 21.4	197.6 ± 11.2
p-value (controls)	***p*=0.019**	*p*=0.322	-
p-value (ORF15)	-	*p*=0.668	***p*=0.019**
IRT 3° (µm)	174.0 ± 26.5	167.7 ± 16.6	174.8 ± 12.4
p-value (controls)	*p*=0.796	*p*=0.343	-
p-value (ORF15)	-	*p*=0.436	*p*=0.796
IRT 1° (µm)	89.0 ± 31.6	62.7 ± 11.9	65.7 ± 13.7
p-value (controls)	***p*<0.001**	*p*=0.757	-
p-value (ORF15)	-	***p*=0.011**	***p*<0.001**
PRC 7° (µm)	89.5 ± 18.8	100.8 ± 10.6	115.3 ± 7.3
p-value (controls)	***p*<0.001**	***p*=0.017**	-
p-value (ORF15)	-	***p*=0.041**	***p*<0.001**
PRC 5° (µm)	96.5 ± 19.3	107.3 ± 10.5	122.6 ± 9.1
p-value (controls)	***p*<0.001**	***p*=0.006**	-
p-value (ORF15)	-	*p*=0.061	***p*<0.001**
PRC 3° (µm)	106.1 ± 23.9	118.8 ± 11.4	133.9 ± 11.5
p-value (controls)	***p*<0.001**	***p*=0.030**	-
p-value (ORF15)	-	*p*=0.080	***p*<0.001**
PRC 1° (µm)	134.9 ± 32.4	151.4 ± 16.9	165.6 ± 15.1
p-value (controls)	***p*<0.001**	*p*=0.160	-
p-value (ORF15)	-	*p*=0.116	***p*<0.001**
